# HIV among pregnant women in Moshi Tanzania: the role of sexual behavior, male partner characteristics and sexually transmitted infections

**DOI:** 10.1186/1742-6405-3-27

**Published:** 2006-10-17

**Authors:** Sia E Msuya, Elizabeth Mbizvo, Akhtar Hussain, Jacqueline Uriyo, Noel E Sam, Babill Stray-Pedersen

**Affiliations:** 1 Department of International Health, Institute of General Practice and Community Medicine, University of Oslo, Norway; 2 Department of Obstetric and Gynaecology, Rikshospitalet University Hospital, University of Oslo, Norway; 3 Kilimanjaro Christian Medical Centre, P.O Box 3010, Moshi, Tanzania

## Abstract

**Background:**

Women continue to be disproportionately affected by HIV in Tanzania, and factors contributing to this situation need to be identified. The objective of this study was to determine social, behavioral and biological risk factors of HIV infection among pregnant women in Moshi urban, Tanzania. In 2002 – 2004, consenting women (N = 2654), attending primary health clinics for routine antenatal care were interviewed, examined and biological samples collected for diagnosis of HIV and other sexually transmitted/reproductive tract infections.

**Results:**

The prevalence of HIV was 6.9%. The risk for HIV was greater among women whose male partner; had other sexual partners (adjusted odds ratio [AOR], 15.11; 95% confidence interval [CI], 8.39–27.20), traveled frequently (AOR, 1.79; 95% CI, 1.22–2.65) or consumed alcohol daily (AOR, 1.68; 95% CI, 1.06–2.67). Other independent predictors of HIV were age, number of sex partners, recent migration, and presence of bacterial vaginosis, genital ulcer, active syphilis and herpes simplex virus type 2.

**Conclusion:**

Development of programs that actively involve men in HIV prevention is important in reducing transmission of HIV in this population. Further, interventions that focus on STI control, the mobile population, sexual risk behavior and responsible alcohol use are required.

## Background

The HIV epidemic continues to take its greatest toll in sub-Saharan Africa, where more than 60% of the world's 40 million infected persons live [1]. Tanzania, a country with a population of 34.5 million is among the worst affected, having 7% of the adults infected with HIV [2]. There is a diverse pattern of trends in HIV prevalence for different geographical areas in the country. In some areas the reports show a decreased trend in the prevalence and incidence of HIV, especially among individuals aged 15–24 years [3,4]. In others, there is a gradual and continuing spread of HIV [4,5]. In all areas however, women continue to experience higher rates of prevalence and incidence than men [2-5], and 58% of the HIV-infected in the whole country are women [6]. There is therefore a need to elucidate risk factors continuing to contribute to the HIV epidemic among women of reproductive age.

In this study we report social, behavioral and biological determinants for HIV, among pregnant women in Moshi urban, Tanzania, including male partner's characteristics and behavior. The study is part of a prospective cohort study that aimed to describe the acceptability of HIV perinatal interventions at the primary health care level as well as to determine factors associated with incident HIV and sexually transmitted infections (STIs) in the postpartum period. The information on determinants for HIV is intended to contribute in improving counseling and in planning for future preventive activities.

## Results

Ninety nine percent of the 2664 women counseled agreed to participate. Of the 2654 participating women, 99 agreed to undergo serological testing but declined gynecological examination. The age of the women ranged from 14–43 years (mean, 24.6 years, standard deviation = 5.4 years) and parity from 0–9 (mean, 1.2). Most were married or cohabiting (91%), had completed 7 years of formal education (79%) and were not formally employed (95%). The average income per month was low; 29% had no income and 65% had an income of less than 30,000 Tanzanian shillings or ~ 30 USD per month. The duration of residence in Moshi ranged from less than a year to 42 years (mean, 12.7 years, median, 10 years). Condom use was low; 75% reported they had never used a condom, with only 13% reporting consistent use.

One hundred and eighty four women were HIV seropositive, giving a prevalence of 6.9% (95% CI, 5.9%–7.9%). The prevalence of HIV increased from 2.8% among women under 20 years to 10.1% in women aged 25–29 years and 8.5% among 35–39 year olds (*P *for trend <0.001), see table 1. In the univariate analysis being single, divorced or separated was more strongly associated with HIV than being married (OR = 1.67), or being in a polygamous relationship compared to those who were not (OR 2.80). Women who consumed alcohol either occasionally or daily had a higher HIV prevalence (OR 1.71) than those who did not. Recent migrants i.e. those who had resided in Moshi for ≤2 years were more likely to be HIV positive than long term residents (*P *for trend 0.009). Other covariates that significantly increased the HIV risk in the univariate analysis were sexual debut at ≤15 years, perception of high risk for HIV and a higher number of lifetime sexual partners (*P *for trend <0.001). No association was found between HIV and religion, employment, education level, income or report of frequent traveling by the women.

**Table 1:** The association between HIV and sociodemographic, sexual behavior and risk perception among 2654 pregnant women in Moshi Tanzania.

**Predictor**	**N**	**(%)**	**% HIV positive**	**Unadjusted OR (95% CI)**		**P value**
**Age (completed years)**						
14 – 19	471	(17.7)	2.8	1		
20 – 24	996	(37.5)	5.9	2.22	1.20 – 4.09	0.01
25 – 29	664	(25.0)	10.1	3.95	2.16 – 7.25	<0.001
30 – 34	382	(14.4)	9.2	3.55	1.85 – 6.82	<0.001
35 – 39	117	(4.4)	8.5	3.29	1.41 – 7.71	0.006
40 +	24	(0.9)	0.0	-	-	
**Years of residence in Moshi**						
3 +	2095	(78.9)	6.4	1		
1 – 2 years	391	(14.7)	7.7	1.22	0.81 – 1.84	0.35
<1 year	168	(6.3)	11.9	1.98	1.20 – 3.26	0.007
**Alcohol consumption**						
No	1833	(69.1)	5.8	1		
Occasionally/weekly	770	(29.0)	9.2	1.66	1.21 – 2.26	0.002
Daily	51	(1.9)	13.7	2.59	1.14 – 5.89	0.02
**Marital status**						
Married	1624	(61.2)	6.0	1		
Cohabiting	790	(29.8)	8.1	1.39	1.00 – 1.93	0.05
Single/separated/divorced	240	(9.1)	9.6	1.67	1.04 – 2.69	0.04
**Polygamy relationship †**						
No	2245	(84.6)	6.0	1		
Yes	296	(11.2)	15.2	2.80	1.95 – 4.02	<0.001
**Number of pregnancies**						
1^st ^pregnancy	968	(36.4)	3.5	1		
2^nd ^pregnancy	700	(26.4)	9.0	2.72	1.77 – 4.17	<0.001
3^rd ^or more	986	(37.1)	8.8	2.66	1.17 – 3.99	<0.001
**Age at first sex (years)**						
19 +	1068	(40.2)	5.7	1		
16 – 18 years	1208	(45.5)	7.0	1.25	0.89 – 1.76	0.19
9 – 15 years	378	(14.2)	10.1	1.85	1.21 – 2.82	0.005
**Number of lifetime sexual partners**						
1	1490	(56.1)	2.3	1		
2	834	(31.4)	9.8	4.53	3.02 – 6.79	<0.001
3	237	(8.9)	17.7	8.95	5.58 – 14.37	<0.001
4 +	93	(3.5)	26.9	15.28	8.66 – 26.97	<0.001
**Casual partner in the past 12 months**						
No	2537	(95.6)	6.7	1		
Yes	117	(4.4)	11.1	1.73	0.95 – 3.14	0.07
**Ever used a condom**						
No	1984	(74.8)	6.0	1		
Yes	670	(25.2)	9.7	1.68	1.23–2.31	0.001
**Perceived risk of HIV infection**						
No risk	891	(33.6)	5.6	1		
Small risk	1006	(37.9)	7.4	1.34	0.92 – 1.94	0.13
Moderate risk	119	(4.5)	10.1	1.89	0.97 – 3.66	0.06
High risk	45	(1.7)	17.8	3.64	1.61 – 8.22	0.002
Don't know	593	(22.3)	6.7	1.22	0.79 – 1.87	0.37

^† ^113 women excluded because currently they do not have a steady partner

Table 2 depicts the univariate analysis of woman's risk of HIV in relation to male partner characteristics. The partners were older by a mean of 6 years. Their age ranged from 17–71 years (mean, 30.6 years, median, 30 years). The risk for HIV in women increased as the partners age increased, (*P *for trend <0.001). E.g. 9.5% of women with partners aged 35–71 were HIV positive compared to 2.8% in those whose partner were <25 years. Further, as the age difference between couples increased to >10 years, so did the likelihood of the women being HIV positive. Women who were aware that their partners had women outside the relationship had the greatest risk for HIV (OR 22.57). Women were also more likely to be HIV infected if they had partners who consumed alcohol (OR 1.71), traveled frequently (OR 1.86), were involved in tourism or the mining industry (OR 4.51), or verbally or physically abused them (OR 1.66). Neither the partner's education nor circumcision was associated with HIV infection.

**Table 2:** Predictors for HIV infection among pregnant women in Moshi, Tanzaniain relation to male partners characteristics

**Predictor**	**N**	**(%)**	**% HIV positive**	**Unadjusted OR (95% CI)**		**P value**
**Partners age (years)^¶^**						
<25	464	(17.5)	2.8	1		
25–34	1425	(53.7)	6.5	2.42	1.34 – 4.37	0.003
35–71	677	(25.5)	9.5	3.62	1.97 – 6.66	<0.001
**Age difference (male – female) in years^¶^**						
0	91	(3.4)	5.5	1		
^-^11 – ^-^1	81	(3.1)	8.6	1.63	0.49 – 5.34	0.42
1 – 10	2047	(77.1)	5.8	1.05	0.42 – 2.64	0.91
11 – 41	347	(13.1)	11.5	2.24	0.86 – 5.85	0.09
**Partner has other women outside the relationship †**						
No	944	(35.6)	2.3	1		
Yes	200	(7.5)	35.0	22.57	13.51–37.69	<0.001
Do not know	1397	(52.6)	6.3	2.82	1.75 – 4.53	<0.001
**Partner consumes alcohol**						
No	1239	(51.3)	5.5	1		
Occasionally/weekly	763	(31.6)	7.6	1.42	0.99 – 2.04	0.06
Daily	411	(17.0)	11.7	2.28	1.55 – 3.36	<0.001
No response	241	(9.1)	4.1	0.75	0.38 – 1.47	0.39
**Partner travel frequently (≥4 times/month)**						
No	1794	(74.3)	6.0	1		
Yes	619	(25.7)	10.7	1.86	1.35 – 2.57	<0.001
No response	241	(9.1)	4.1	0.68	0.35 – 1.311	0.25
**Partner's occupation**						
Professional	81	(3.1)	4.9	1		
Driver	282	(10.6)	6.4	1.31	0.43 – 3.99	0.63
Army/police force/security guard	265	(10.0)	12.1	2.64	0.91 – 7.71	0.07
Tour guide/miner	58	(2.2)	19.0	4.51	1.36 – 14.97	0.02
Others ^#^	828	(74.2)	6.0	1.24	0.45 – 3.44	0.68
**Verbal or physical abuse by partner**						
No	2062	(77.7)	6.6	1		
Yes	351	(13.2)	10.5	1.66	1.13 – 2.43	0.01
No response	241	(9.1)	4.1	0.61	0.32 – 1.17	0.14
**Partner as 1^st ^person wished to share HIV results with**						
Yes	2390	(90.1)	6.1	1		
No	264	(9.9)	14.4	2.58	1.76 – 3.79	<0.001

¶ 88 women excluded because they do not know their partners age.

† 113 women excluded because currently they do not have a steady partner.

# Farmer, trader, technical and unskilled labor

A history of treatment for sexually transmitted infection (STI) symptoms and the presence of laboratory confirmed infection were strongly associated with HIV, table 3. The presence of genital ulcers (*P *= 0.003), bacterial vaginosis (*P *< 0.001), gonorrhoea (*P *= 0.03), active syphilis (*P *= 0.001), and herpes simplex type 2 (*P *= 0.003) increased the risk for HIV in the univariate analysis.

**Table 3:** The association between HIV-1 with genital symptoms, clinical signs and sexually transmitted infections among pregnant women in Moshi Tanzania.

**Predictor**	**N**	**(%)**	**% HIV positive**	**Unadjusted OR (95% CI)**		**P value**
**Treatment for STI symptoms in past 12 months^¶^**						
No	1969	(74.2)	5.9	1		
Yes	685	(25.8)	9.8	1.72	1.25 – 2.35	0.001
**Report abnormal vaginal discharge or itch at interview**						
No	2200	(82.9)	6.1	1		
Yes	454	(17.1)	10.8	1.85	1.31 – 2.61	<0.001
Genital ulcer on examination*						
No	2514	(94.7)	7.0	1		
Yes	41	(1.5)	19.5	3.24	1.47 – 7.12	0.003
Bacterial vaginosis*						
No	2022	(76.2)	5.7	1		
Yes	533	(20.1)	12.8	2.43	1.77 – 3.33	<0.001
Trichomoniasis*						
No	2428	(91.5)	7.0	1		
Yes	127	(4.8)	10.2	1.52	0.84 – 2.75	0.171
Candidiasis*						
No	2264	(85.3)	6.9	1		
Yes	291	(11.0)	8.9	1.32	0.85 – 2.03	0.214
Gonococcal infection (GND)*						
No	2542	(95.8)	7.1	1		
Yes	13	(0.5)	23.1	3.94	1.07 – 14.43	0.04
**Active syphilis**						
No	2631	(99.1)	6.8	1		
Yes	23	(0.9)	26.1	4.86	1.89 – 12.49	0.001
**Herpes simplex virus type 2 ^†^**						
No	844	(31.8)	12.3	1		
Yes	427	(16.1)	18.5	1.62	1.17 – 2.22	0.003

GND = Intracellular gram-negative diplococci on cervical smear.

^¶ ^Vaginal discharge, genital itch, genital ulcer, dysuria, dyspareunia.

*Missing values because 99 women were not examined and/or samples not provided.

^† ^Test done on 1271 women only

In the multivariate analyses (table 4), the most significant determinant for HIV was having a partner with women outside the relationship [AOR = 15.11 (CI, 8.39–27.20)]. Other independent predictors of HIV were age ≥20 years, sexual debut at ≤15 years, ≥2 lifetime sexual partners, residing in Moshi ≤2 years, a male partner who consumed alcohol daily, a partner who was away >4 times/month, the presence of genital ulcer during examination, bacterial vaginosis, active syphilis and HSV-2.

**Table 4:** Multivariate analyses of predictors for HIV infection among pregnant women in Moshi Tanzania.

**Predictor**	**Adjusted OR (95% CI) ^†^**		***P *value**
**Age (years)**			
14 – 19	1		
20 – 24	2.45	(1.19 – 5.07)	0.015
25 – 29	4.77	(2.27 – 10.03)	<0.001
30 – 34	3.92	(1.74 – 8.86)	0.001
35 – 39	3.73	(1.29 – 10.81)	0.02
**Years of residence in Moshi**			
3 +	1		
1 – 2 years	2.23	(1.36 – 3.66)	0.002
<year	2.49	(1.26 – 4.91)	0.008
**Number of lifetime partners**			
1	1		
2	3.29	(2.10 – 5.17)	<0.001
3	4.08	(2.33 – 7.14)	<0.001
4 or more	6.11	(2.97 – 12.57)	<0.001
**Age at first sex (years)**			
19 +	1		
9 – 15 years	1.81	(1.06 – 3.11)	0.03
**Partner has women outside the relationship**			
No	1		
Don't know	2.70	(1.60 – 4.57)	0.001
Yes	15.11	(8.39 – 27.20)	<0.001
**Partner consumes alcohol**			
No	1		
Daily	1.70	(1.06 – 2.67)	0.03
**Partner travel frequently (≥4 times/month)**			
No	1		
Yes	1.79	(1.22 – 2.65)	0.003
**Partner's occupation**			
Professional	1		
Army/police/security guard	2.56	(0.62 – 10.57)	0.19
Tour guide/miner	3.02	(0.79 – 15.11)	0.11
**Partner as 1^st ^person wished to share HIV results with**			
Yes	1		
No	1.71	(1.03 – 2.84)	0.04
**Genital ulcer at examination**			
No	1		
Yes	2.92	(1.07 – 7.94)	0.03
**Bacterial vaginosis**			
No	1		
Yes	2.00	(1.36 – 2.95)	<0.001
**Active syphilis**			
No	1		
Yes	4.41	(1.22 – 15.95)	0.02
**HSV-2^¶^**			
No	1		
Yes	1.36	(1.01 – 1.98)	0.04

^† ^Adjusted for all the variables in the table plus marital status, polygamy, number of pregnancies, history of STIs, report of vaginal discharge/itch and male partners age and report of verbal or physical abuse.

^¶ ^Adjusted for all the variables in the table

## Discussion

Nearly 7% of the women in the study were HIV-positive, indicating that HIV is still a major public health problem among women of reproductive age in Moshi urban. The prevalence observed (6.9%), is similar to the prevalence of 7.3% described among women aged 15–49 years in Kilimanjaro region, in the recent Tanzania HIV/AIDS Indicator survey [2]. Given the high antenatal attendance rates in the area (>97%), women attending antenatal clinic can be used as a sentinel surveillance population in monitoring trends of HIV infection among adults aged 15–49 years, despite its known limitations [1,7].

The HIV prevalence was greater among women who started sex at an early age (≤15 years). The prevalence peaked early at 10% among 25–29 year olds [2,5,8]. This suggests that most infections in women occur at a younger age, during the first few years after sexual debut [8]. Immature genital tract and cervical ectopy which is common in young women might increase the risk [9,10]. Untreated STIs may magnify the biological susceptibility [8,11]. Further, because women tend to have older partners at debut or later, they might be at higher risk because they might be exposed to previously infected partners [8,12-14]. Preventive programs should therefore target young people, especially women, with the aim to empower them to delay sexual debut and to improve their negotiating skills, especially regarding condom use.

Male factors were strong predictors for HIV. Having a partner who had other women outside the relationship increased the HIV risk by 15-fold. Alcohol use by the partner also increased the HIV risk. The better economic and cultural position of men compared to women in most African settings leads to a skewed balance of power in sexual relationships [12-15]. Men are thus the main decision makers of when and under what circumstances sex will take place [12,15,16]. Several reports show that married men report more casual partnerships than married women [8,12,13], and when they use alcohol, they have increased risk of unprotected sex and commercial sex [17]. However, due to women's lower social and cultural position than men, women's economic dependence, and domestic violence, most are not in a position to negotiate safe sex [12,15,16,18]. In this study women who gave a history of physical or verbal abuse by the current partner had both an increased risk of HIV and of not coming back for their HIV test results [18,19]. It is thus vital to design programs that actively involve men in HIV preventive interventions and in other reproductive health issues. The focus of preventive efforts should be to encourage men to use condoms consistently in any sexual encounter with a person of unknown HIV status and reduce the numbers of sexual partners. There is also a need to promote the use of voluntary counseling and testing services as a preventive tool especially for people entering into stable partnerships. Further, culturally sensitive interventions that address domestic violence should be integrated in HIV preventive programs [15,16,18].

Women with partners who were mobile (i.e. frequent travelers, or involved in tourism or the mining industry) had a higher HIV prevalence. Mobile men have been shown to report more sexual risk behavior, (e.g. multiple partners, excess alcohol intake and sex with commercial sex workers), putting them and consequently their partners at risk of HIV [20]. It may also be that women with absent partners are more likely to engage in casual partnerships because they are either free, lonely, or experience economic hardship. Recently, a study among couples in Mwanza, Tanzania, showed that there is an increase of sexual risk behavior in both the mobile person and the partner staying behind [21]. Further work is required to assess the vulnerability of this special group of women who are partners of mobile men and preventive efforts extended to both the mobile partners and their women.

A higher HIV prevalence was observed in women who had recently migrated into Moshi (≤2 years). Compared to women who had resided in Moshi for >3 years, they were younger than 25 years (78% vs 49%; *p *= < 0.001), had no or incomplete primary education (14% vs 10%; *p *= 0.006), had no income (39% vs 26%; *p *< 0.001), reported more casual partners in the past 12 months (8.3% vs 4.1%; *p *= 0.01) and had more GUD (2.6% vs 1.3%; *p *= 0.04). It may be that most of these women, who had moved to an urban area to seek a better life, had to engage in high risk behavior in order to survive, as shown in South Africa [22]. Mobility and internal migration seems to be an important character of the HIV epidemic in Moshi. Long term programs that will identify migrant women and promote safer sex and economic empowerment are required.

Genital ulcer, active syphilis and HSV-2 were independent risk factors for HIV. STIs increase the efficiency of HIV transmission [11]. Genital ulcers increase the HIV susceptibility by disruption of the mucosa barrier, thus providing an easy port of entry and increase the recruitment and activation of HIV susceptible inflammatory cells. The inflammation and ulceration increases HIV shedding in the genital tract, thus the HIV infectiousness [10,11]. HSV-2 and syphilis are ulcerative STIs, and are highly prevalent among women in resource poor settings [8,22-24]. Effective management of STIs reduces the HIV incidence [25], therefore STI control should be prioritized. One strategy for reaching more women will be an integration of STI management in reproductive health clinics. Further, because a growing number of ulcers are caused by HSV-2 [26], its management should be integrated in the GUD syndromic guidelines in Tanzania. Bacterial vaginosis has been shown to be strongly associated with HIV [27]. It is known to be the most common cause of vaginal discharge and consistent correlation between the symptom of vaginal discharge and BV warrants the use of a syndromic approach for timely treatment of this infection [11,27]. Prompt treatment will reduce not only the risk of HIV transmission, but also the adverse obstetric and gynecological complications associated with BV [11,27,28].

Sporadic use of condoms did not confer protection to HIV, similar to what was observed elsewhere [13,29]. Condoms are effective when used correctly and consistently. Consistent condom use was low among the women. It may also be that people who know or suspect they have HIV may tend to use condoms more to protect their partners. Or condom use may be a marker of high risk sexual behavior as shown in one study, where people with multiple partners reported higher rates of use of condoms than those with a single partner [29].

This study had several limitations. This was a cross-sectional study, so the odds ratios observed may overestimate risk estimates and the associations may not be causal. Secondly, sensitive information regarding the male partner's behavior characteristics was reported by the women. The accuracy may be low due to lack of openness regarding sexual matters between the partners, and probably some degree of guesswork regarding casual partners. Limitation in self-reported data on sexual behavior has been shown, where there is a tendency to under report sexual risk behavior [8,13]. The results observed in this data may thus be an underestimation of the true association between HIV and behavior characteristics. HIV decreases fertility in women, both from sub fertility and from increased early pregnancy loss [30]. HIV infected women also have higher rates of tubal infertility secondary to pelvic inflammatory diseases [31], therefore the prevalence presented might fail to reflect those who are not able to become pregnant. Lastly, women aged ≥35 years were few (5.3%) in the antenatal clinic, therefore the prevalence might not reflect the picture among women of that age in the community [32].

## Conclusion

HIV is still a major health problem among women of reproductive age. The behavior and other characteristics of the male partners in this study were important predictors for HIV in women. Therefore, involvement of men in HIV prevention and in all aspects of reproductive health programs is of the utmost importance if we want to make advances in preventing HIV in women and in the community at large. Empowering women with the skills and rights to negotiate in sexual matters must be more successfully addressed. Other important preventive strategies should aim at control of STIs, reduction of number of partners, increased use of condoms in long term partnerships, responsible alcohol use and targeting mobile people.

## Methods

### Study area, population and study procedures

Moshi urban district is situated in Northern Tanzania, and is one of 6 districts in Kilimanjaro region. It is the capital of the region and has a population of about 230,000 people. Most people are employed in the private sector and the main income generating activities are tourism, trading and agriculture.

The present study was conducted in the two largest primary health care clinics, Majengo and Pasua. These clinics were selected because they have the largest number of patients and represent women from the largest geographical areas (administrative wards). Pregnant women attending the clinics for routine care, who were in their 3^rd ^trimester and residing in Moshi urban, were eligible to participate. They were informed about the study and its aims, and were invited to participate between June 2002 and March 2004. Women wishing to participate in the study signed a written informed consent. For illiterate women the right thumb print was taken as a signature.

Trained research nurses conducted individual pretest counseling of every woman. The women were assured that the information they provided and test results would be treated confidentially and that participation in the study was voluntary. They could withdraw from participation or follow-up at any time and this would not affect their prenatal care or access to other services at the clinic. Ten women refused to participate after the pretest session while 2654 women agreed to participate. Interviews were then conducted in a private room to obtain information on socio-demographic variables, sexual behavior, obstetric history, perceived risk of HIV, alcohol use and on current and past sexually transmitted infection (STI) symptoms, by using a standardized pre-tested questionnaire. Detailed information regarding the male partner's demographic and behavioral factors, alcohol use and communication between couples was collected. The interviews were conducted in Kiswahili, the national language.

After the interview, a general and gynecological examination was performed. Genital ulcers, warts and abnormal vaginal discharge were diagnosed clinically during the examination. Vaginal secretions were collected for measuring pH level, whiff test, for Gram-staining and identification of *Trichomonas vaginalis*, and *Candida *species. An endocervical swab was collected for Gram-staining and identification of *Neisseria gonorrhea*. Genital samples were not collected from 99 (4%) of the women because they did not want to undergo speculum examination. Venous blood was collected for serological analysis of HIV, syphilis and herpes simplex virus type 2. The women were assigned numeric identifiers and all the questionnaires, follow-up forms and laboratory samples were labeled with matching numbers to maintain confidentiality.

The women were asked to return for their HIV/STIs results in one week. Post-test counseling was conducted individually with each woman, where possible by the same nurse who conducted the pre-test counseling. HIV positive women were given a single dose of Nevirapine (tablet) to take at the onset of labor and they were instructed to bring their children within 72 hours after delivery to receive the Nevirapine syrup. This regimen was for prevention of mother-to-child transmission (PMTCT) of HIV according to the HIVNET 012 regimen [33]. Genital infections diagnosed during clinical examination were treated syndromically based on the Tanzanian Ministry of Health guidelines. Laboratory confirmed infections were treated a week later, during the post test visit. All women were encouraged to inform their partners and bring them for counseling and testing, and those with proven sexually transmitted infections were given a contact card to give to their partners so that they could come for treatment. All the services were free of charge for both the women and their partners. At the time the study was conducted, there was no routine service for counseling and testing of pregnant women for HIV, nor was there a PMTCT program. Permission for the study was obtained from the Tanzanian Ministry of Health and the Norwegian Ethical Committee.

### Laboratory procedures

Except for HSV-2 testing, which was performed at the laboratory at KCMC referral hospital, all other tests were performed at the clinics. Within 6 hours of collection, blood was centrifuged on site and serum was tested for HIV by using two rapid tests, Determine HIV 1/2 (Abbott Laboratories, IL, USA) and Capillus HIV1/2 (Trinity Biotech, Ireland). HIV was diagnosed when both the test results were positive. In case of discordance between the two tests, a third test, the ELISA test, Vironostika HIV Uni-form II (Organon Teknika, Boxtel, Netherlands) was used. Seven samples were discordant by the rapid tests. Three of the seven samples tested positive by the third test and were diagnosed positive, the remaining four were negative. Active syphilis was diagnosed by positive results of both the rapid plasma reagin test (RPR; Becton Dickinson, MD, USA) and a specific test, Determine Syphilis TP (Abbott Laboratories, IL, USA). HSV-2 was detected by the type-specific HSV-2 ELISA (Focus Diagnostics, Cypress, California USA).

A wet mount of the vaginal swab was prepared in normal saline for microscopic identification of motile *Trichomonas vaginalis*, yeast cells and for presence of clue cells. Direct microscopy was done on Gram-stained genital swabs for the detection of leucocytes, *Candida *species and gram-negative diplococci. The diagnosis of bacterial vaginosis was made according to the Amsel criteria [34]. Candidiasis was diagnosed by visualization of *Candida *species on wet mount or gram-stained vaginal swabs.

### Statistical analysis

The data were analyzed using SPSS statistical software, version 10.0 (SPSS, Chicago, IL, USA). Statistical comparison between groups was made using χ^2 ^and odds ratios (OR) were calculated with a 95% confidence interval (CI) to measure the strength of association between potential predictor factors and HIV. Multiple logistic regression was executed to adjust for potential confounders. Variables were entered in the models based on the level of significance in the univariate analyses at *P *< 0.20 or if they were known to be important risk factors for HIV based on previous reports. Stepwise procedure using an automated backward selection model was used to determine a final model. The level of significance was set at *P *≤ 0.05.

## Abbreviations

STIs: sexually transmitted infections

HSV-2: herpes simplex virus type 2

GUD: genital ulcer disease

PMTCT: prevention of mother-to-child transmission of HIV

OR: odds ratio

CI: confidence interval

## Competing interests

The author(s) declare that they have no competing interests.

## Authors' contributions

SEM: Designed the study, coordinated recruitment of patients, collected and entered data, analyzed data and drafted the manuscript.

EM: Designed the study, participated in data analysis, and reviewed the drafted manuscript.

AH: Designed the study, interpreted and analyzed the data, reviewed the drafted manuscript.

JU: Participated in data collection, laboratory testing and data analysis, also reviewed the drafted manuscript.

NES: Designed the study, supervised laboratory testing, reviewed the drafted manuscript.

BSP: Designed and coordinated the study, interpreted the data, reviewed the drafted manuscript.

All the authors read and approved the final manuscript.
